# A Small Molecule Stabilizer of the MYC G4-Quadruplex Induces Endoplasmic Reticulum Stress, Senescence and Pyroptosis in Multiple Myeloma

**DOI:** 10.3390/cancers12102952

**Published:** 2020-10-13

**Authors:** Snehal M. Gaikwad, Zaw Phyo, Anaisa Quintanilla Arteaga, Sayeh Gorjifard, David R. Calabrese, Daniel Connors, Jing Huang, Aleksandra M. Michalowski, Shuling Zhang, Zheng-Gang Liu, John S. Schneekloth, Beverly A. Mock

**Affiliations:** 1 Laboratory of Cancer Biology and Genetics, National Cancer Institute, NIH, Bethesda, MD 20892, USA; snehal.gaikwad@nih.gov (S.M.G.); zaw@jhu.edu (Z.P.); aquintanillaarte@luc.edu (A.Q.A.); sgorji@uw.edu (S.G.); dgconnor@buffalo.edu (D.C.); huangj3@mail.nih.gov (J.H.); michaloa@mail.nih.gov (A.M.M.); zhangsh@mail.nih.gov (S.Z.); 2 Chemical Biology Laboratory, National Cancer Institute, NIH, Frederick, MD 21702, USA; dave.calabrese@nih.gov; 3 National Center for Advancing Translational Sciences (NCATS), NIH, Rockville, MD 20850, USA; 4 Laboratory of Immune Cell Biology, National Cancer Institute, NIH, Bethesda, MD 20892, USA

**Keywords:** MYC G4-quadruplex stabilizer, endoplasmic reticulum stress, senescence, pyroptosis, inflammasome, caspase 1, gasdermin D, NLRP3, ASC and pannexin 1

## Abstract

**Simple Summary:**

The DNA G-quadruplex (G4) present in the promoter of the MYC oncogene, commonly amplified in cancers, including multiple myeloma, represents a potential anti-cancer target. A previously identified MYC G4-stablizer, which demonstrated cytotoxicity and senescence in myeloma cells, was discovered to induce endoplasmic reticulum stress and non-apoptotic cell death, pyroptosis. Cancers including myeloma escape apoptosis through upregulation of anti-apoptotic proteins and drug resistance; therefore, induction of pyroptosis provides an alternate therapeutic option. Thus, our study provides a disease-specific experimental strategy for identifying new investigational drugs in cancer treatment.

**Abstract:**

New approaches to target MYC include the stabilization of a guanine-rich, G-quadruplex (G4) tertiary DNA structure in the NHE III region of its promoter. Recent screening of a small molecule microarray platform identified a benzofuran, D089, that can stabilize the MYC G4 and inhibit its transcription. D089 induced both dose- and time-dependent multiple myeloma cell death mediated by endoplasmic reticulum induced stress. Unexpectedly, we uncovered two mechanisms of cell death: cellular senescence, as evidenced by increased levels of p16, p21 and γ-H2AX proteins and a caspase 3-independent mechanism consistent with pyroptosis. Cells treated with D089 exhibited high levels of the cleaved form of initiator caspase 8; but failed to show cleavage of executioner caspase 3, a classical apoptotic marker. Cotreatment with the a pan-caspase inhibitor Q-VD-OPh did not affect the cytotoxic effect of D089. In contrast, cleaved caspase 1, an inflammatory caspase downstream of caspases 8/9, was increased by D089 treatment. Cells treated with D089 in addition to either a caspase 1 inhibitor or siRNA-caspase 1 showed increased IC_50_ values, indicating a contribution of cleaved caspase 1 to cell death. Downstream effects of caspase 1 activation after drug treatment included increases in IL1B, gasdermin D cleavage, and HMGB1 translocation from the nucleus to the cytoplasm. Drug treated cells underwent a ‘ballooning’ morphology characteristic of pyroptosis, rather than ‘blebbing’ typically associated with apoptosis. ASC specks colocalized with NLRP3 in proximity ligation assays after drug treatment, indicating inflammasome activation and further confirming pyroptosis as a contributor to cell death. Thus, the small molecule MYC G4 stabilizer, D089, provides a new tool compound for studying pyroptosis. These studies suggest that inducing both tumor senescence and pyroptosis may have therapeutic potential for cancer treatment.

## 1. Introduction

The proto-oncogene MYC is often deregulated in ~70% of human cancers and over-expressed in multiple myeloma (MM) [1,2]. MYC functions as a pleiotropic transcription factor and regulates gene expression of pathway members involved in cell growth, proliferation, replication, metabolism, and apoptosis [3]. In MM pathogenesis, MYC activation and overexpression results from translocation, rearrangement or gain of the MYC locus, thus, it is an attractive target for myeloma therapy [2,4,5,6,7,8]. The transcriptional regulation of MYC involves multiple promoters, enhancers and transcriptional start sites. The upstream P1 promoter of c-MYC contains a nuclease hypersensitivity element (NHE) III that controls 85–90% of the transcriptional activation of this gene [9]. This NHE III region contains a purine (guanine) rich non-coding strand that can form a non-canonical, Hoogsteen-bonded structure referred to as a G-quadruplex (G4). The G4 functions as a transcriptional repressor element and MYC transcription can be controlled by ligand mediated G4 stabilization. In general, G4 structures exist in dynamic equilibrium with their normal duplex counterparts and do not naturally form at high frequency to prevent transcription, in part because they can be resolved by helicases [9,10,11]. Extensive efforts to develop small molecules that bind and stabilize the MYC G4 have unlocked a potential method for targeting MYC driven cancers [11,12,13,14,15,16]. Pan G4-stabilizing ligands, such as pyridostatin and TMPyP4 have been associated with inducing DNA damage leading to senescence or apoptosis [17].

Previously, we demonstrated a mechanism for inhibiting MYC function by stabilizing the G-quadruplex (G4) present in the MYC promoter region and consequently inhibiting transcription [14]. Using a small molecule microarray, we found a selective MYC-G4 binding drug with a benzofuran scaffold (D089) that not only inhibited MYC expression in myeloma cell lines, but also selectively induced G1 arrest in MYC-driven cancer cell lines containing the MYC-G4 sequence. Here, we show that D089 induces the canonical pathways involved in the unfolded protein response, endoplasmic reticulum stress, and inflammatory responses to modulate cell cycle progression, senescence and caspase 1-mediated cell death by pyroptosis.

Pyroptosis is a pathway of programmed cell death (PCD) characterized by activation of inflammatory responses [18,19]. Initially discovered in the context of host cell death during microbial infections [20], this form of cell death shares characteristics with apoptosis, but the distinct activation of caspase 1 differentiates it from the canonical apoptotic pathway. Although pyroptosis is associated with chromatin condensation, in general the nuclear morphology remains intact, while DNA fragmentation is not necessarily observed in cells undergoing this process [21,22]. The activated pyroptotic inflammatory sensors NLRP3, AIM2 and pyrin recruit pro-caspase 1 with the help of apoptosis-associated speck-like protein containing a CARD domain (ASC) [23], an adaptor protein which consists of a pyrin domain (PYD) and a caspase recruitment domain (CARD). Pyroptosis is frequently mediated by the protease-inflammatory caspases (-1, -4 and -5 in humans and -11 in mice) leading to the activation of inflammation [24,25]; it is characterized by assembly of inflammasomes, formation of large structures called pyroptosomes and pore formation in the cell membrane [20,26,27,28,29,30]. Pyroptosomes are an aggregation of ASCs and pro-caspase-1, which allows processing of caspase-1 into two active forms (p10 and p20) leading to secretion of potent endogenous pyrogenic molecules: interleukin-1β (IL-1β) and IL-18 inflammatory cytokines as well as high mobility group box 1 proteins (HMGB1) [31,32]. Caspase 1 and/or -11 are responsible for proteolytic cleavage of gasdermin-D (53kDa) to N-terminal (31kDa) and C-terminal (22kDa) fragments; the N-terminal fragment targets and permeabilizes cell membranes forming pores, and promoting secretion of mature IL-1β [32,33,34,35].

## 2. Results

### 2.1. D089, a Small Molecule Stabilizer of the MYC-G4 Quadruplex Reduces MYC Transcription and Induces Cytotoxicity in Myeloma Cells

**Figure 1:**
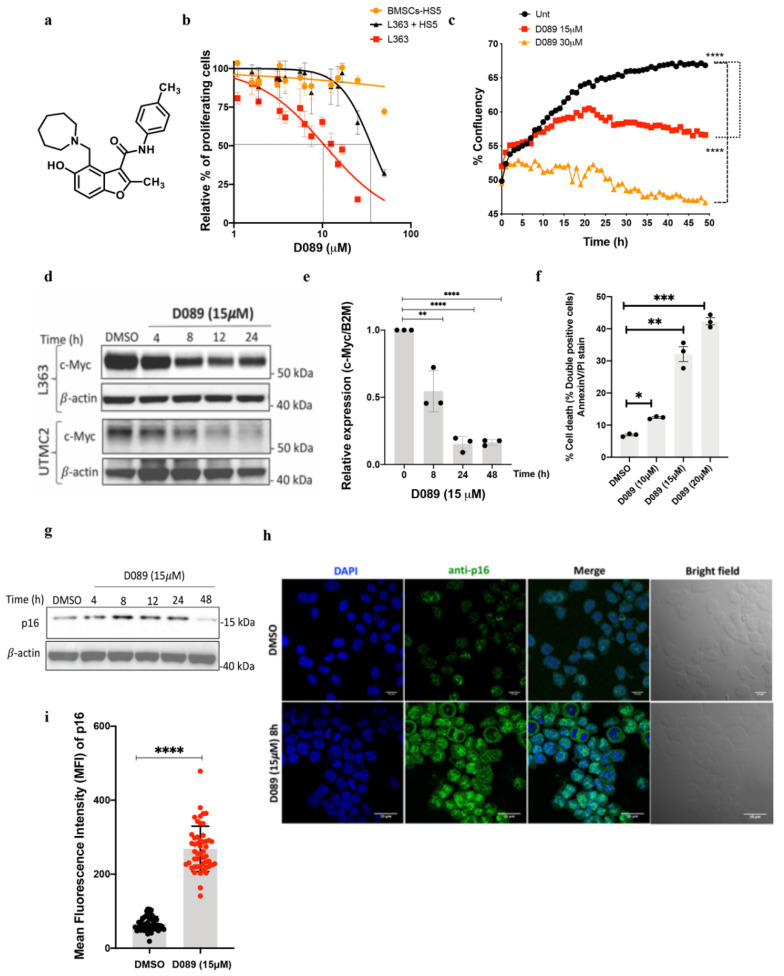
D089 silences MYC expression and induces cytotoxicity in multiple myeloma cells. (**a**) Chemical structure of benzofuran-containing compound D089. (**b**) Dose response of multiple myeloma cell line L363 in co-culture with BMSCs after 48 h treatment with D089. The data is presented as percentage of viable cells relative to untreated cells. (**c**) Cell proliferation of L363 cells in a 48 h time course after D089 treatment with 15 or 30 μM; measured by IncuCyte as percent confluent (**** *p* < 0.0001). (**d**) MYC protein expression over 24 h in L363 (15 μM) and UTMC2 (25 μM) cells after D089 treatment. The uncropped Western blots have been shown in Table S2. (**e**) Quantitative PCR analysis of MYC mRNA expression in L363 cells over 48h following D089 treatment. Each bar graph represents the average percent ± SD (*n* = 3) (** *p* = 0.01; **** *p* < 0.0001) relative to untreated cells. (**f**) Dose-dependent cell (L363) death (annexin-V, PI staining by flow cytometry) after 48 h treatment with D089. The average percent of apoptotic cells from three replicates are indicated. Each bar graph represents the average percent ± SEM (*n* = 3) (* *p* = 0.01; ** *p* = 0.02; *** *p* < 0.0001) relative to untreated cells. (**g**) Immunoblotting of p16 in L363 cells after D089 treatment for a 48 h time course. (**h**) L363 cells were treated with 15 μM D089 for 8h and p16 protein expression was analyzed by confocal microscopy (p16 in green) counter stained with DAPI for nuclei (blue). Scale bars: 10 µm; Magnification: 63× objective lens. (**i**) Bar graph indicating the mean fluorescence intensity (MFI) of p16 staining calculated by the (Fiji) ImageJ software from 50 cells taken from three different images.

Investigation of a focused library of drug-like small molecules that bind MYC G4 sequences identified D089 (compound **1**) [14] (Figure 1a) which selectively binds and stabilizes a G4 structure located in the MYC promoter. Previously, D089 was shown to be cytotoxic to MM cell lines with minimal effect on the viability of peripheral blood mononucleocytes or cells lacking a G4 in the MYC promoter [14]. In this study, we performed time course experiments of cells treated with D089 to measure L363 cell viability and MYC protein and transcript levels (Figure 1b–f). D089 induces cytotoxicity in L363 MM cells either alone [half inhibitory concentration (IC_50_) = 13 (11–16) µM or in co-culture with HS-5 bone marrow stromal cells (BMSCs) [IC_50_ = 35 (28–43) µM] without affecting the BMSCs-HS5 alone (IC_50_ = < 50 µM) (Figure 1b). Similar responses were seen in AMO1 and UTMC2 MM cells (Figure S1a,b). D089 significantly decreased cell proliferation in a time and dose responsive manner (Figure 1c), which correlated with a gradual decrease in MYC protein expression seen over a 24-h time course in MM cell lines treated at their IC_50_ (Figure 1d). Quantitative RT-PCR analysis of MYC transcript levels shows a decrease in MYC expression over 48 h in L363 MM cells with D089 treatment (Figure 1e). Dose-dependent increases in annexin V+/7AAD+ cells were seen in L363 cells when compared to vehicle (DMSO) treated cells indicating that decreases in cell viability led to increased cell death. Cells treated with 10 μM D089 increased double positive (annexin V/PI) staining from 7% to 12.3% in 48 h, while treatment with 15 μM showed 33% double positive staining (Figure 1f, Figure S1c). Previously, increases in β-gal staining with treatment indicated that a proportion of the MM cells were undergoing senescence [14]. In our current study, western blot and confocal analyses of drug-treated L363 cells immunostained for p16, a known senescence marker, also showed significant increase in p16 protein expression by 8h after drug treatment (Figure 1g–i). Similarly, p21 and γ-H2AX protein expression levels also increased consistent with induction of cellular senescence and DNA damage (Figure S1d,e).

In order to validate the specificity of D089, c-MYC was over-expressed by a constitutive CMV promoter (lacking a G4 in its promoter region) and treated with D089 in HEK293T cells. A cytotoxic dose response shows that these HEK293T cells are relatively resistant to D089 treatment showing an IC_50_ of 50 μM (Figure S1f). Similarly, transient overexpression of c-MYC by CMV-c-MYC-IRES-GFP (293T_MYC) demonstrated high expression of c-MYC as compared to endogenous untransfected 293T or 293T cells with transient transfection with control CMV-IRES-GFP (293T_GFP) vector (Figure S1g). Interestingly, no significant difference in expression of c-MYC or senescence associated p16 was observed upon D089 treatment (50 μM) at 48 h (Figure S1g). Overall, the results indicate that D089 reduces MYC expression and induces cytotoxicity by stabilizing the G4 in the MYC promoter. To further examine potential off-target effects of D089, A Burkitt’s lymphoma cell line, CA46, containing a chromosomal translocation in the MYC locus disrupting G4 regulation, was used. As previously reported by Boddupally et al. [36] and Felsenstein, et al. [14], CA46 cells did not show changes in proliferation after treatment with MYC G4 stabilizers. We performed a time course experiment with CA46 cells treated with D089 (15 μM) and protein expression changes were monitored over 24 h. As expected, MYC levels did not change. We also did not observe any changes in expression of Caspase 3 or Caspase 1, indicating limited cell death (Figure S2a–d).

### 2.2. Drug Treatment Modulates the ER Stress Pathway in Myeloma

**Figure 2:**
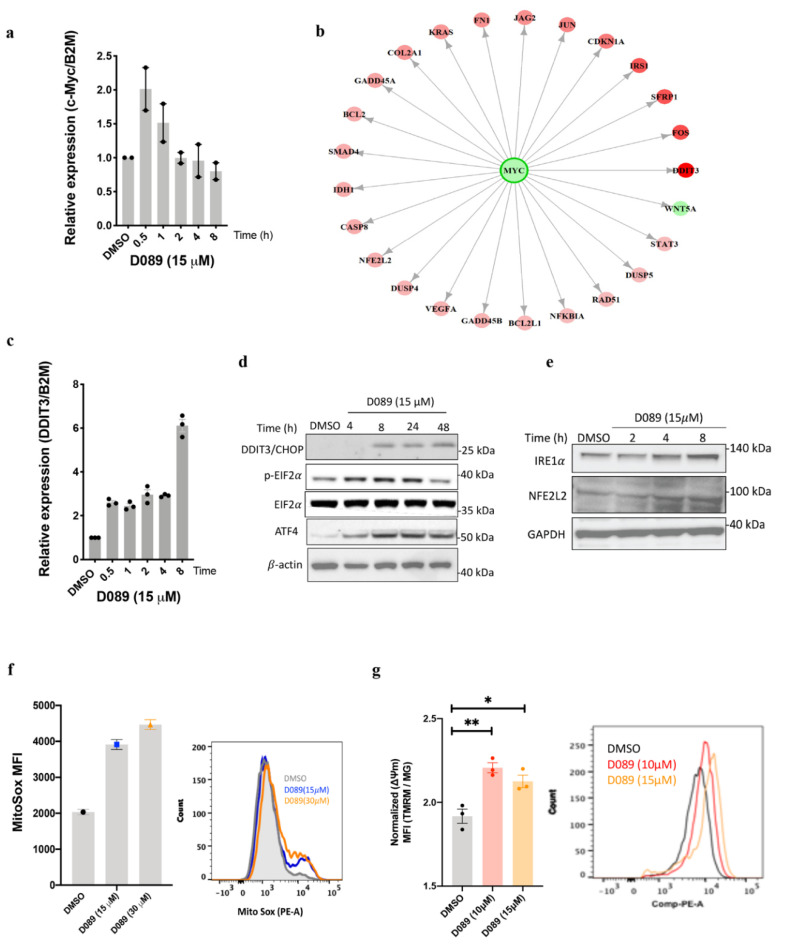
D089 treatment induces endoplasmic reticulum stress and unfolded protein response. (**a**) q-PCR analysis of MYC mRNA expression upon D089 treatment in an 8 h time-course. (**b**) Differentially regulated genes (IPA analysis of Nanostring data) after 8 h of D089 treatment. Genes significantly upregulated (red) and significantly down regulated (green) by D089 treatment are shown. (**c**) q-PCR analysis of DDIT3 mRNA expression after D089 treatment in an 8 h time-course. (**d**,**e**) Immunoblotting of down-stream effectors of ER stress pathway after D089 treatment over time. (**f**) A representative histogram of the mean fluorescence intensity (MFI) indicative of mitochondrial reactive oxygen species by MitoSox dye after 2 h of D089 treatment. (Mean ±SEM absorbance by flow cytometric analysis). (**g**) D089 treatment for 48 h increaed MFI of TMRM dye, denoting enhanced mitochondrial membrane potential. The data was normalized by MFI of MG used as a counter stain. Each bar graph represents the fold change of MFI (TMRM/MG) (*n* = 3) relative to untreated cells (* *p* = 0.02; ** *p* = 0.001).

In our previous study [14], NanoString (770 gene Cancer Panel) analyses were employed to identify early response genes and affected signaling pathways after drug treatment. Changes in gene expression over time (0.5 h, 1 h, 2 h, 4 h and 8 h) were assessed in L363 cells treated with D089 (15 µM). As previously reported, MYC mRNA levels decreased over time, despite a very early increase in mRNA levels over the first hour (Figure 2a). Following further analysis of the Nanostring data, sixty-five genes were found to be differentially expressed after treatment with D089 for 8 h (early response genes). D089-responsive genes were selected using two criteria at the 8th hour of treatment: normalized gene expression greater than 14 (50th percentile threshold) in either the DMSO or the D089 sample, and at least a 1.7 log2 fold change (70th percentile threshold) in expression (Table S1). Ingenuity Pathway upstream regulator analysis of genes showing at least a 1.7-fold change in gene expression (Figure 2b) were enriched in MYC targets (64 molecules were affected by MYC; Fisher’s exact test, *p* < 0.001) which was consistent with on-target effects. Additionally, reactome pathway analysis of the D089 affected genes using the Gene Ontology–PANTHER analysis platform, identified 5 top pathways (Table 1) involved in endoplasmic reticulum (ER) stress, apoptosis, inflammation (inflammasome) and cell cycle regulation, suggesting possible mechanisms of drug action.

The most upregulated gene at the 8 h time point was DDIT3/CHOP, a transcription factor which induces cell death in response to ER stress as a result of unfolded or mis-folded proteins (Table S1, Figure 2b). NanoString data for DDIT3 was validated by RT-qPCR of RNA samples from myeloma cells following D089 treatment. Since DDIT3/CHOP promotes endoplasmic reticulum (ER) biosynthesis by dephosphorylating eIF2a [37,38] in stressed cells, we also examined the protein expression of downstream effectors of ER stress induced by the unfolded protein response (UPR). Upregulation of DDIT3 (Figure 2c,d) was followed by increased phosphorylation of eIF2a, and NFE2L2 (NRF2) and subsequent upregulation of ATF4, all effectors of the ER pathway (Figure 2d,e, Figure S2e,f). Since stressed cells frequently have high levels of reactive oxygen species (ROS), MitoSox dye staining of drug-treated cells indicated a dose dependent increase in mitochondrial ROS levels after 2 h (Figure 2f). A 48 h treatment with D089 at increasing concentrations showed enhanced mitochondrial membrane potential (MMP) (ΔΨm) by tetramethyl rhodamine methyl ester (TMRM) dye assays followed by normalization with Mito Tracker Green (MG) staining (Figure 2g), ultimately leading to cell death.

### 2.3. D089 Cytotoxicity Is Independent of Initiator and Effector Caspases

**Figure 3:**
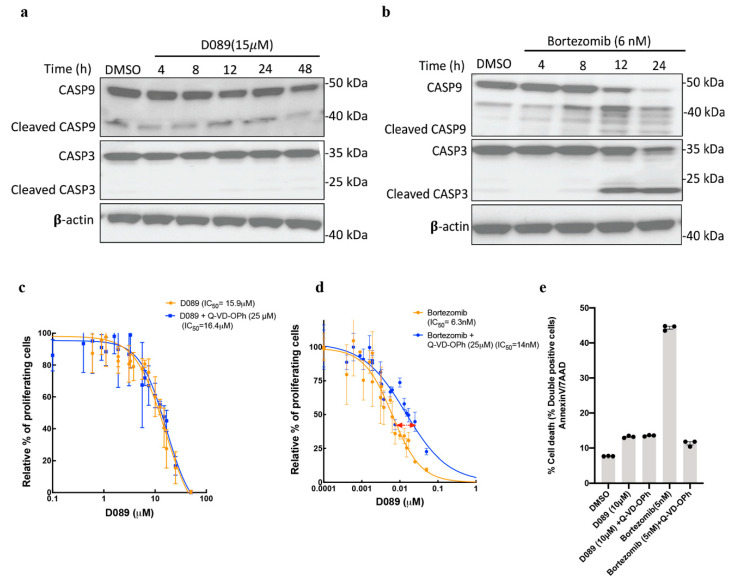

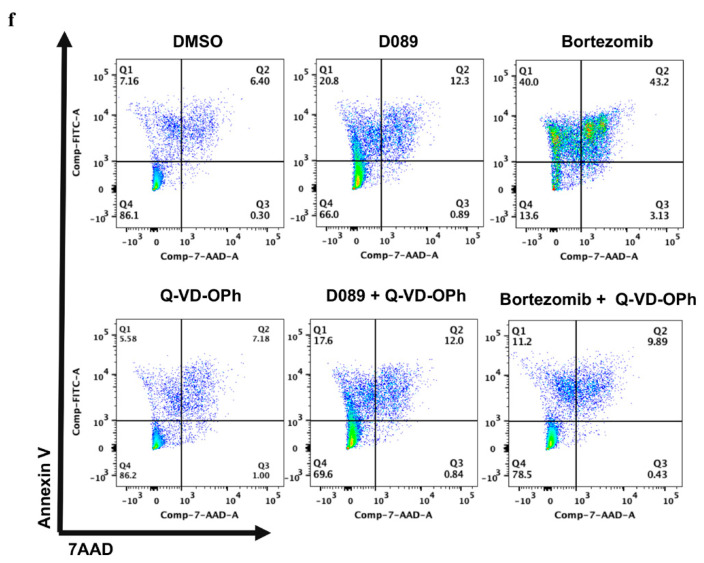
D089 kills cells independent of effector caspase 3 (CASP3). (**a**,**b**) Immunoblotting of full length CASP9 and CASP3 and their cleaved forms after D089 (15 μM) (**a**) or bortezomib (5 nM) (**b**) treatment over time. (**c**,**d**) Dose-response curves of L363 cells after 48 h of treatment with D089 (**c**) or Bortezomib (**d**) along with co-administration of the CASP3 inhibitor Q-VD-OPh (25 nM). A shift in IC50 concentration of bortezomib was observed (marked with a red arrow). (**e**,**f**) L363 cells were stimulated with D089 (10 μM) for 48 h and cell death was assessed with annexin-V and 7-AAD staining followed by flow cytometry. Quantification of the percent cell death by D089 and bortezomib after co-administration of Q-VD-OPh (**e**); the double-positive staining in quadrant 2 (Q2) represents the percentage of cell death (**f**).

**Figure 4:**
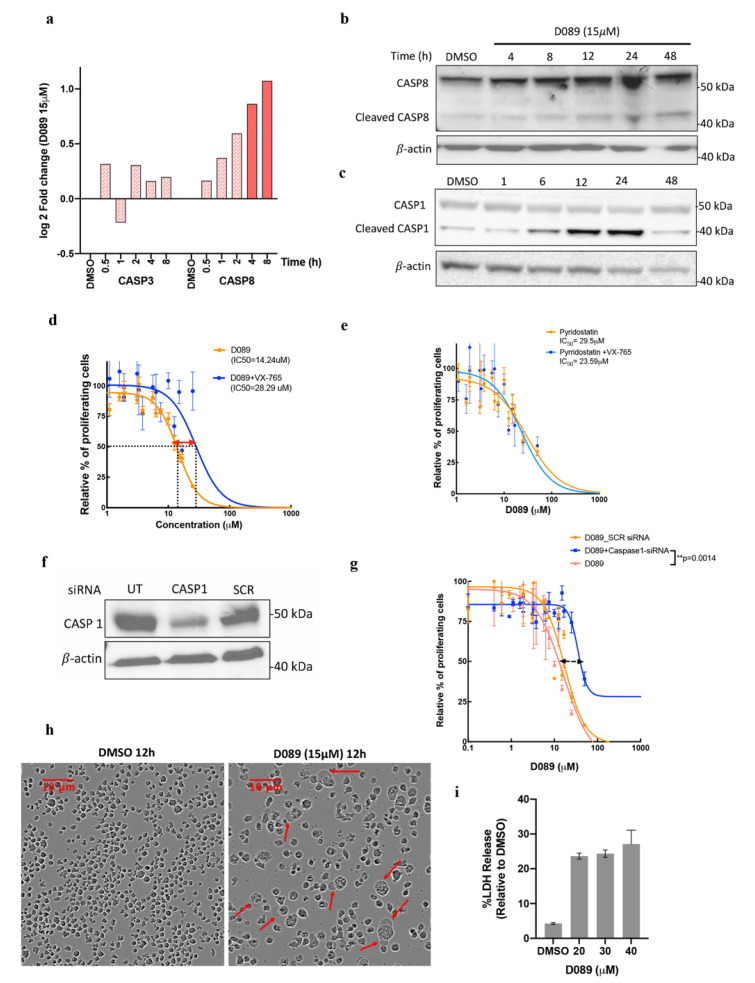
D089 induces caspase-1 and 8 dependent cell death mechanism (pyroptosis). (**a**) Gene expression levels of Caspase-3 (CASP3) and Caspase-8 (CASP8) in L363 cells from NanoString analysis after D089 treatment in an 8h time course compared to DMSO (vehicle control). The dark red shaded bar represents a log2-fold increase of at least 1.7 in CASP8 gene expression at 4 and 8 h; light shading reflects log2-fold change <1.7. (**b**,**c**) Immunoblotting of CASP8 (b) and CASP1 (**c**) after D089 (15 μM) treatment in L363 cells over 48 h. Cleaved products in both indicate active protein. (**d**,**e**) Dose-response curves of L363 cells after 48 h treatment with D089 (**d**) or the pan-G4 inhibitor, pyridostatin (**e**) along with co-administration of the Caspase-1 specific inhibitor, VX-765 (40 nM) (**f)** Immunoblotting for CASP1 expression in L363 cells treated with CASP1-siRNA and scrambled (SCR) siRNA as control for 48 h, (UT = Untransfected). (**g**) Dose response of L363 cells after 48h treatment with D089 along with co-administration of CASP1 si-RNAs or a scrambled (SCR) siRNA control. A shift in D089 IC_50_ concentration was observed in D089 + Caspase-1 siRNA (*p* = 0.001). (**h**) ‘Ballooning’ morphology indicates membrane disruption (red arrows) in L363 cells following D089 treatment by IncuCyte live-cell imaging. (**i**) Assessment of lactose dehydrogenase activity (LDH) in cell free supernatants of L363 cells treated with increasing concentrations of D089 after 2 h. The values shown are the mean ± standard error of the mean (*N* = 3).

To elucidate the cell death mechanisms, we examined the protein expression of initiator caspase-9 (CASP9) and executioner caspase-3 (CASP3) along with their cleaved products (indicators of apoptosis) following treatment. Surprisingly, D089 had a modest effect on CASP9 cleavage, but failed to induce cleavage products of CASP3, indicating that “classical apoptosis” is not likely to be the primary cell death mechanism (Figure 3a). As a positive control, L363 cells were treated with bortezomib, a proteasome inhibitor known to kill cells via accumulation of misfolded proteins and CASP3 cleavage indicative of apoptosis. Unlike D089, treatment with bortezomib induced robust cleavage of CASP9 and CASP3, confirming canonical apoptotic cell death upon treatment (Figure 3b). Further evidence that caspases 3 and 9 did not play a major role in mediating D089-induced cell death was obtained by co-administration of D089 with Q-VD-OPh, a pan-caspase apoptosis inhibitor. Similar experiments with bortezomib were also performed as a positive control for Q-VD-OPh activity. Co-administration of Q-VD-OPh failed to alter D089 induced cytotoxicity (Figure 3c); in contrast, the caspase 3/9 inhibitor partially rescued cell death induced by bortezomib (Figure 3d). In keeping with these observations, mRNA levels of caspase-3 remained comparable to untreated control samples over an 8h period of D089 treatment (Figure 4a). Thus, caspases (3 and 9) involved in classical apoptosis were not major players in D089 induced cell death. Of note, two anti-apoptotic genes, BCL2 and BCLXL, were upregulated following D089 treatment (Figure 2b) and may also contribute to this phenotype. Similar results were obtained by monitoring the percent cell-death by co-administration of Q-VD-OPh (25 μM) with either D089 (10 μM) or Bortezomib (5 nM) (Figure 3e,f).

### 2.4. D089 Induces Caspase-8 and -1 Dependent Cell Death Mechanism-Pyroptosis

Time-dependent increases in caspase-8 (CASP8) mRNA levels were observed after D089 treatment; similar increases were seen in protein expression of pro- and cleaved CASP8 in L363 (Figure 4a,b and Figure S3a and AMO1 cells (Figure S3b). Activation of the executioner caspase-8 cleaves either pro-caspase-3 or pro-caspase-1 into their active cleaved products [39,40]. Since CASP3 was not cleaved after treatment, we next examined caspase-1 (CASP1) protein expression and found a steady increase in cleaved caspase-1 over 24h (Figure 4c, Figure S3c).

Further evidence that cell death was CASP1 dependent was found when L363 cells were treated simultaneously with VX-765, a CASP1 specific inhibitor, and D089. The IC_50_ doubled (from 14 to 28 μM), suggesting partial protection from cell death via CASP1 inhibition (Figure 4d). When this experiment was repeated with pyridostatin, a drug known to stabilize a large number of quadruplexes throughout the genome, the CASP1 inhibitor did not alter its toxicity (Figure 4e). This indicates that unlike D089, pyridostatin induced cell-death is CASP1 independent. Transient knockdown of CASP1 (Figure 4f) using RNAi similarly doubled the IC50 of D089 and therefore, further validated that D089 triggered cell death in a CASP1 dependent manner (Figure 4g). We also monitored morphological changes in the L363 cells by incucyte analysis. Notably, after 12 h of D089 treatment, the dying cells became swollen with large singular bubbles extending out from the plasma membrane (Figure 4h). This single large membrane ‘bubble’ phenotype, coupled with CASP1 cleavage, is associated with pyroptosis. In contrast, CASP3 cleavage results in membrane ‘blebbing’ or ‘petals’ typically associated with apoptosis [41].

Pyroptosis, but not apoptosis, is associated with loss of membrane integrity associated with cell death and can be assessed by lactate dehydrogenase (LDH) activity. We observed an increase of LDH release after treatment with D089, relative to DMSO treated cells, suggesting that D089 treatment disrupts the membrane integrity (Figure 4i).

### 2.5. D089 Triggered Pyroptosis by Priming the Inflammasome Signaling Pathway

**Figure 5:**
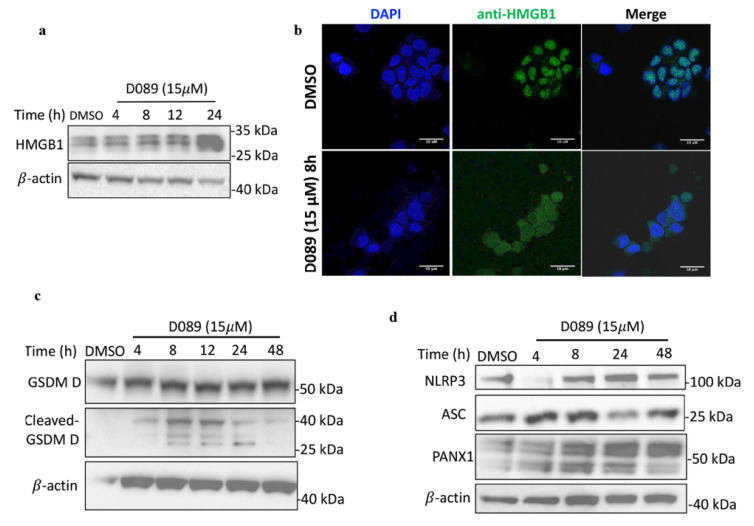

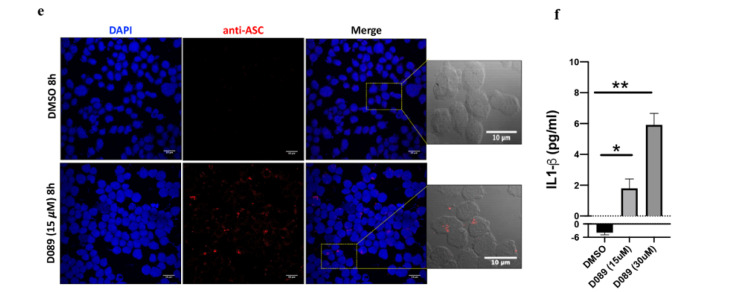
D089 triggers pyroptosis by priming inflammasome signaling. L363 cells were treated with DMSO or D089 (15µM) for the indicated times. (**a**) Immunoblotting of HMGB1; (**b**) Confocal imaging by immunofluorescence for HMGB1 protein expression. D089 induced HMGB1 translocation from the nucleus to the cytoplasm. Scale bars: 10 µm; Magnification: 63× objective lens. (**c**,**d**) Protein expression of gasdermin D (**c**) and the pyroptosis markers, NLRP3, ASC and PANX1 (**d**) after D089 treatment. Each experiment was performed independently. (**e**) Confocal imaging of cells treated for 8h, fixed and stained for ASC protein expression. A dot/speck is formed in the cytoplasm of D089 treated cells compared with ASC expression in DMSO treated cells. Inset demonstrates the ASC staining (red) in contrast to the bright field. Scale bars: 10 µm; Magnification: 63× objective lens. (**f**) Soluble IL1-β levels from supernatant of cultured cells (ELISA (pg/mL concentration ± standard error of the mean (*n* = 3) (* *p* < 0.05; ** *p* < 0.01)) treated with DMSO or two different concentrations of D089 for 8 h.

Observations of ER stress and ROS production (Figure 2), pyroptotic cell death (Figure 4), and enrichment of the NLRP inflammasome pathway (Table 1 and Table S1) in response to drug treatment led us to investigate the cellular and molecular events in this pathway in more depth. Stressed cells often release proteins known as damage-associated molecular patterns (DAMPs) such as HMGB1 (high mobility group B -1) [42]. We observed a gradual increase in HMGB1 protein levels (Figure 5a) with D089 treatment over time as well as its translocation from the nucleus to the cytoplasm (Figure 5b). Once in the cytoplasm, HMGB1 can activate nuclear factor NFκB and increase its translocation from the cytoplasm to the nucleus [43,44,45]. Interestingly, NF-κB is an important transcription factor for gasdermin D (GSDMD) [46], which has been shown to be essential for pyroptosis and is cleaved by CASP1 [25]. We found that D089 treatment induced an increase in cleaved (active) GSDMD (Figure 5c, Figure S3b).

GSDMD and CASP1 are both found in the ROS-activated multiprotein inflammasome complex associated with NLRP3 (nucleotide-binding oligomerization domain receptors (NLR) pyrin domain-containing 3) [47,48,49]. NLRP3, as well as other pyroptotic markers that play a role in formation and activation of inflammasomes, namely the adaptor protein ASC (apoptosis-associated speck-like protein containing a caspase activation and recruitment domain), and PANX1(pannexin-1) [50], which forms pores at the plasma membrane [51] also showed an early increase in expression with D089 treatment over time (Figure 5d,e).

CASP1 is recruited to the inflammasome by ASC, and contributes to the maturation and processing of IL1-β [47]. IL-1β release, from supernatants of drug treated myeloma cells increased significantly after 8h (Figure 5f), as did ASC (Figure 5d,e) which is also assembled into a large protein complex known as a ‘speck’ (Figure 5e), and constitutes the pyroptosome.

**Figure 6:**
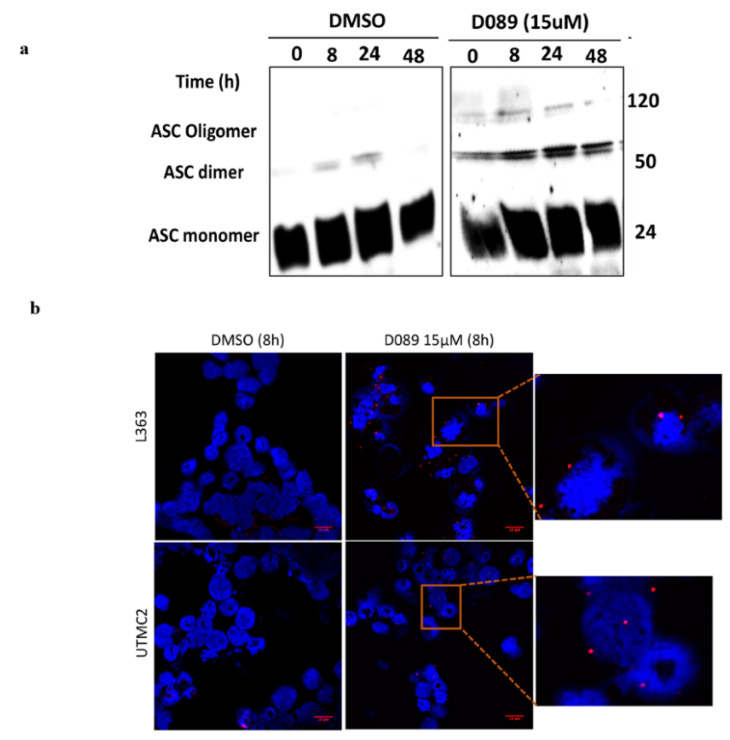

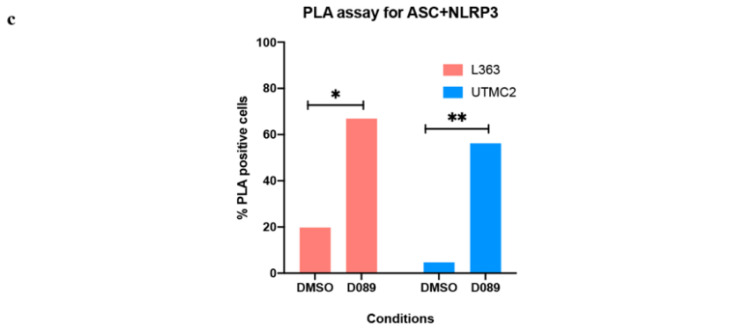
D089 induces pyroptosome and inflammasome complexes. (**a**) Immunoblot of ASC oligomers in lysates from L363 cells treated with DMSO or D089 (15 μM) for 8, 24 and 48 h. A native PAGE gel was used to detect ASC oligomers. (**b**) Representative confocal images of PLA signals for ASC and NLRP3 in L363 cells after DMSO or D089 (15 μM, 8 h) treatment (cells are counter-stained with DAPI). Inset in the D089 treated panel indicates the presence of PLA signals. Scale bars: 10 µm; Magnification: 63× objective lens. (**c**) Quantitative analysis of PLA positive cells: percent of total nuclei ± standard error (*n* = 150 cells from five different images) (* *p* < 0.05; ** *p* < 0.01) counted after DMSO or D089 (15 μM, 8 h) treatment.

As a signature event of pyroptosis is inflammasome and pyroptosome formation, we analyzed whether D089 caused ASC oligo-formation to establish these complexes by native PAGE and immunoblotting. We observed the formation of ASC dimers and oligomers in myeloma cells as early as 8h after treatment (Figure 6a). 

Further, we performed proximity ligation assays (PLA) using ASC antibody in conjunction with NLRP3 antibody. In two separate myeloma cell lines, we observed significant PLA signals for ASC colocalized with NLRP3 after 8 h of D089 treatment (Figure 6b,c). Taken together, all of these results support contributions of both senescence and pyroptosis, in ER stress-induced myeloma cell death by the MYC G4 stabilizer, D089).

## 3. Discussion

In the human genome, formation of dynamic G4 structures in the promoter regions of several oncogenes, including MYC, has led to the development of novel, small molecules able to specifically bind and stabilize these G4 motifs to block transcription and stimulate anti-tumor activity [10,11,36,52,53,54,55]. In our previous study, the MYC G4 ligand D089, a benzofuran scaffold compound, induced G1-cell cycle phase arrest and senescence as a result of MYC repression, yet a detailed mechanism of cell death was not determined [14].

D089 treatment of three independent MM cell lines either alone or in co-culture with HS5- BMSCs to mimic the myeloma microenvironment [56] was cytotoxic either alone or in co-culture with BMSCs. D089 did not induce cytotoxicity in the HS-5-BMSCs alone, indicating specificity in anti-cancer activity. Induction of senescence by D089 was shown previously by b-gal staining [14]. In the current study, high expression of p16, a cellular senescence marker, peaked by 8 h. Consistent with senolytic activity and increased p16 expression, increases in p21 and γ-H2AX (an indicator of DNA damage), were also observed.

During MM pathogenesis, a network of stress mechanisms including endoplasmic reticulum (ER) stress and the unfolded protein response (UPR) and are frequently activated by enhanced antibody production [28,57,58]. These pathways and others involved in cell death, inflammation and cell cycle regulation were altered in myeloma cells treated with D089 after 0.5, 1, 2, 4 and 8 h. MYC gene expression levels showed an initial increase followed by a marked decrease at later time points. Concomitant increases in gene expression were seen in c-jun and c-fos, both early response genes involved in cell cycle regulation. A rapid increase in gene expression was observed in DDIT3 (DNA-damage induced transcript 3), a member of the UPR pathway and potent inducer of ER stress mediated cell death. Notably, each of these genes have MYC binding sites in myeloma cells [59]. Upregulation of the UPR pathway was evident through increased protein expression of downstream effectors, p-EIF2α, ATF4, and NFE2L2 (NRF2). Myeloma cells treated with D089 also produced higher levels of reactive oxygen species (ROS) and experienced disruption of their mitochondrial membrane potential, additional hallmarks of ER stress. The convergence of ER signals can result in both the activation of inflammasomes [27,35,60,61,62,63] and the upregulation of DDIT3 which encodes C/EBP homologous protein (CHOP), a transcription factor which commits the cell to PCD [64,65].

**Figure 7:**
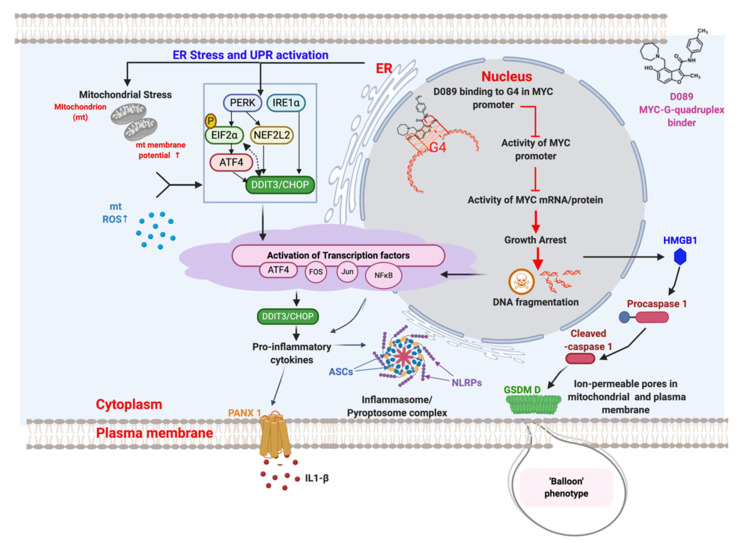
Schematic representation of signaling events associated with D089 induced cell death by pyroptosis. D089 binds to the MYC promoter in the nucleus resulting in growth arrest and DNA fragmentation. Simultaneously, endoplasmic reticulum stress leads to the activation of several transcription factors triggering inflammatory cytokines and formation of inflammasome complexes. Activation of caspase 1 and Gasdermin D results in a ‘balloon’ phenotype that marks the swelling in the cell. These observations are consistent with pyroptosis as the mechanism of programmed cell death. This figure was created using *BioRender* software.

To our surprise, CASP3 cleavage, often associated with apoptosis, was not involved in mediating PCD upon treatment with D089. CASP3 cleavage was not observed after drug treatment and a dual pan-caspase inhibitor failed to affect D089-induced cell death in myeloma cells. Time dependent increases in CASP8 gene expression, as well as CASP8 and CASP1 protein cleavage, led us to investigate a role for D089 in inducing pyroptosis, an alternate form of PCD. Inhibition of CASP1, either by a small molecule inhibitor or by siRNA, diminished the effect of D089 in myeloma cells. Caspase-1 activation can result in rapid formation of membrane pores, significant cell swelling followed by membrane rupture, and release of intracellular content, such as LDH and interleukins, into extracellular spaces [66]. Indeed, treatment with D089 resulted in all of these responses (Figure 7). Interestingly, CA46 BL cells bearing MYC translocations treated with D089 did not show increases in CASP1 cleavage indicating that the binding of D089 to the MYC G4 is important for its cytotoxic activity.

Microscopy of myeloma cells treated over a 48 h period revealed that D089 induced a cell morphology resembling a “balloon” protruding from the cell as early as 12 h after treatment. This change in morphology was similar to that seen in studies demonstrating membrane pore formation to mediate cellular lysis during pyroptosis [39], and is distinct from canonical apoptotic phenotypes. Further molecular evidence of pyroptotic pathways included activation of inflammasome formation resulting in cleavage of GSDMD and/or increased signaling of PANX1. Both effects have been shown to trigger membrane pore formation during pyroptosis [34,49,67] (Figure 7).

Consistent with CASP1 activation and the ballooning phenotype, a signaling cascade of HMGB1 translocation from the nucleus to the cytoplasm was observed in D089 treated myeloma cells. HMGB1 translocation is a DAMP signal for inflammasome activation [68,69]. D089 triggered an increase in NLRP3, ASC and ASC specks which are key components of inflammasome/ pyroptosome complex formation. In fact, proximity ligation assays confirmed the co-localization of ASC specks with NLRP3 in myeloma cells. Thus, inflammasome/pyroptosomes activation and cleavage of GSDMD and PANX1 are likely to induce pore formation leading to the “ballooning” morphology and the subsequent release of LDH and IL-1b seen in the drug treated myeloma cells. These studies demonstrate a connection between ER stress activation, cellular senescence, and the induction of CASP1/NLRP3 inflammasome mediated pyroptotic cell death in myeloma cells treated with the D089 G4 stabilizer (Figure 7).

## 4. Materials and Methods

### 4.1. Cell Culture and Culture Conditions

Human multiple myeloma (MM) cell lines L363, UTMC2, AMO1 and the CA46 BL cell line were obtained and characterized as previously described [70]. HS5-BMSCs were obtained from ATCC. HEK293T and HS5 cells were cultured in DMEM and MM cells were cultured in Advanced RPMI1640 supplemented with 10% heat-inactivated fetal bovine serum, 2 mM L-glutamine, 100 mg/mL penicillin and streptomycin 100 U/mL, (Gibco, Life Technologies, Gaithersburg, MD, USA and incubated at 37 °C with 5% CO_2_. The authenticity of cell lines was confirmed with CNV Fingerprint Multiplex PCR Reaction (Multiplex PCR kit (#206143), Qiagen, Germantown, MD, USA.)

### 4.2. Drug Compounds

For in vitro studies, D089, G-quadruplex binding stabilizer was generated and used as previously described [14]. Pan-caspase inhibitor, Q-Vd-OPh and caspase-1 inhibitor, VX-765 were purchased from Sigma-Aldrich. Bortezomib was purchased from LC labs. Drugs were dissolved in DMSO (Sigma, St. Louis, MO, USA) to 10 mmol/L and stored at −80 °C. Pyridostatin was procured from Cayman Chemicals (Ann Arbor, MI, USA). For in vitro studies, drugs were added to cells and washed 2× with cold PBS before downstream applications.

### 4.3. Plasmid Transfections

HEK293-T cells (0.25 × 10^6^/well) were seeded into 6-well plates and allowed to attach overnight. pMY-CMV-MYC-IRES-GFP and pMY-CMV-IRES-GFP (Cell Biolabs’ RTV-021) [11] (CMV, cytomegalovirus promoter driven plasmid) were transiently transfected using Lipofectamine 3000 (ThermoFisher, Waltham, MA, USA). Briefly, plasmid DNA was diluted in OptiMEM Reduced Serum Media (ThermoFisher) along with P3000 reagent and incubated at room temperature (RT) for 5 min. Simultaneously, Lipofectamine 3000 reagent was also diluted in OptiMEM for 5 min. Diluted DNA was added in diluted lipofectamine reagent in a 1:1 ratio followed by incubation at RT for 20 min. The DNA-lipid complex was added to cells with gentle mixing and incubated at 37 °C overnight. OptiMEM media was removed and replaced with complete growth media containing D089 or DMSO (0.1% final) and incubated at 37 °C for 48 h.

### 4.4. RNA Interference

siRNA transfection was performed following manufacturer’s reverse transfection protocols for DharmaFECT 1 transfection reagent (GE Dharmacon, Lafayette, CO, USA) in a 96-well or 6 well plates. The siRNAs used in the study from Dharmacon Inc. are listed below (Table 2).

Briefly, siRNAs were resuspended in 1× siRNA Resuspension buffer (GE Dharmacon B-002000-UB-100) and used at a final concentration of 50 nM in OptiMEM. Transfection reagents and siRNA were mixed and incubated at 25 °C for 20 min to form siRNA-liposome complexes. L363 cells (0.5 × 10^4^ cells/well (96-well plate) or 0.5 × 10^5^ cells/well (6-well plate)) were added with the transfection mixture; after for 24 h, complete growth media containing D089 or DMSO (0.1% final) were added to each well and incubated at 37 °C for 48 h.

### 4.5. Cell Proliferation (MTS) Assay

Cell proliferation experiments were performed using Cell Titer 96 Aqueous One Solution Cell Proliferation Assay System (Promega, Madison, WI, USA). Briefly, cells were plated in quadruplicate on clear, flat-bottomed 96-well tissue culture treated plates (Corning Costar) and treated with drugs at 37 °C, 5% CO_2_. After incubation, MTS reagent was added directly to the wells and incubated for 90 min at 37 °C, 5% CO_2_. The absorbance of MTS formazan was immediately read at 490 nm (Omega 640 spectrophotometer, (BMG Labtech, Cary, NC, USA). A blank measurement was taken from the absorbance of the wells with media only and subtracted accordingly. Percentage cell viability was normalized to the absorbance of untreated wells and averaged from the four wells. The data were analyzed with GraphPad Prism 8 software (San Deigo, CA, USA) and represented as percent (%) cell growth relative to control.

### 4.6. Total RNA Isolation and Quantitative Real Time RT-PCR

RNA was first isolated from cells using manufacturer’s protocols from the Qiagen RNeasy Mini Kit using QIAshredder columns. RNA concentrations were determined using an ND-1000 spectrophotometer (NanoDrop, Thermo Fisher Scientific, Madison, WI, USA). cDNA was reverse transcribed using random primers and a master mix of MultiScribe^TM^ reverse transcriptase (Applied Biosystems, Thermo Fisher Scientific, Foster City, CA, USA). Master mix was added to 1 µg of RNA and cycled at 25 °C for 10 min, 48 °C for 60 min, 95 °C for 5 min, and finally held at 4 °C until use. cDNA was diluted 1:10 with ultrapure, RNAse free water before use in qPCR. Samples for qPCR were prepared with a 1:4 ratio of diluted cDNA to primer master mix (1× SYBR Green PCR Mix: Applied Biosystems, 0.2 µM forward primer, 0.2 µM reverse primer, ultrapure H_2_O). qPCR was performed on an Applied Biosystems 7500 Fast Real-Time PCR System per manufacturer’s protocol. All primers (Table 3) were validated to ensure the absence of primer-dimers, the presence of a single peak dissociation curve, and an acceptable standard curve from serial dilutions of the same cDNA.

### 4.7. RNA Isolation for NanoString nCounter^®^ Gene Expression

L363 cells were treated with 15 μM of D089 for given (0.5, 1,2, 4, and 8 h) timepoints. RNA was isolated from cells using Trizol reagent (Sigma, St. Louis, MO, USA) and further purified using a Qiagen RNeasy Mini Kit. Isolated RNA was eluted in a 30 μL volume and its purity assessed using a NanoDrop ND-1000 spectrophotometer (OD 260/280 nm 1.7–2.5).

### 4.8. Nanostring nCounter^®^ Gene Expression Quantification

RNA (100 ng) was analyzed by NanoString nCounter XT CodeSet Gene Expression Assays which delivers direct, multiplexed measurements of gene expression through digital readouts of the abundance of mRNA transcripts (Panel Name: PanCancer Pathways RLF: NS_CancerPath_C2535); these data were generated by Felsenstein et al. [14] and further mined in this study. The nCounter XT assay simultaneously measures the expression levels of 730 target genes plus 40 endogenous control (house-keeping) genes in a single hybridization reaction using an nCounter CodeSet. Each assay run includes a reference sample consisting of in vitro transcribed RNAs of six targets that are used for normalization purposes. The raw expression data (counts) generated by the nanoString nCounter^®^ system were imported into nanoString nSolver™ Analysis Software 4.0. Platform specific variability was accounted for with the geometric mean of the four positive controls (ERCC_00117.1, ERCC_00112.1, ERCC_00002.1, and ERCC_00002.1), followed by assay-specific normalization with the geometric mean of six house-keeping genes (PRPF38A, MRPS5, AGK, FCF1, USP39, and EDC3) chosen by the geNorm algorithm. Upon inspection of the distribution of normalized gene expression, the 50th percentile was chosen as the threshold of low expression (signal ≤ 14). Large treatment effects were defined as the 70th percentile of fold differences between treatment and control samples (fold change > 1.7). Genes with low expression levels in all samples or with small treatment effect at all time points were removed, leaving 134 genes for the subsequent analyses. Gene-level treatment effects were analyzed with hierarchical clustering and heatmap display. Pathway enrichment was assessed with gene ontology overrepresentation using Fisher’s exact test and Reactome collection. These analyses and associated visualizations were generated using R programing language (R 3.5.1 and Bioconductor 3.7) and Palantir Foundry platform (https://www.palantir.com/palantir-foundry/). The IPA (Ingenuity Pathway Analysis) Upstream Regulator analysis was carried out for the selected transcriptional targets (Qiagen; www.qiagen.com/ingenuity).

### 4.9. Protein Extraction and Western Blotting

Cell pellets were spun down at 1000 rpm for 5 min following designated treatments and resuspended in RIPA lysis (Sigma-Aldrich, St. Louis, MO, USA) buffer supplemented with protease and phosphatase inhibitors (Chem Cruz, SantaCruz Inc, Dallas, TX, USA) for 1–2 h. The lysates were vortexed and sonicated in an ice water bath for 5 min on high, with intervals of 30 s on, 1 min off. The lysates were then centrifuged at 13,000 rpm for 10 min. Resulting protein lysates were quantified by Pierce^TM^ BCA Protein Assay Kit (ThermoFisher #23225). 30ug of proteins were run on NuPAGE Bis-Tris Gels (ThermoFisher NP0322BOX) at 125–150 V for 1.5h and transferred to nitrocellulose membranes using the iBlot 2 Dry Blotting System (ThermoFisher). Ponceau S (Sigma-Aldrich) staining was used to validate transfer of proteins. The blots were blocked with 10% dry milk for 30 min and washed three times before incubating overnight in 4 °C cold room with primary antibodies dissolved in 5% BSA at concentrations indicated by the manufacturer. Residual primary antibodies were washed away with TBST solution three times for 15 min each prior to incubation with secondary antibodies conjugated to Horseradish peroxidase (HRP) in 5% milk for 1 h at room temperature. Blots were subsequently washed with TBST and submerged in Dura Chemiluminescent Substrate (ThermoFisher) and imaged using the ChemiDoc Touch Imaging System (Bio Rad, Hercules, CA, USA). All primary antibodies used in the studies were either of rabbit or mouse origin. Details of primary and secondary antibodies are listed in Table 4.

#### ASC Oligomerization

Cells were lysed in buffer (20 mM Tris HCl [pH 7.5], 250 mM NaCl, 3 mM EDTA, 3 mM EGTA, 0.5% NP-40). The lysates were vortexed and rotated at 4 °C for 30 min followed by centrifugation (13000 rpm, 10 min). Proteins (30 µg) were loaded on NATIVE-PAGE Bis-Tris Gels gels and proteins were transferred on PVDF membranes and treated as above. Primary ASC antibody (sc-514414; 1:500) and Anti-mouse IgG (Cell Signaling Technologies, Beverly, MA, USA, # 7076, 1:5000) were used to determine the oligomer formation.

### 4.10. Microscopy Imaging

#### 4.10.1. Immunofluorescence

Cells treated with D089 at defined time points were collected and counted by cell counter. 20,000 cells were washed in ice-cold 1× PBS and diluted in 100 mL of cold PBS. The cells were cytospun at 1000rpm for 5 min. The slides were fixed in 100% chilled methanol for 5 min, followed by permeabilization with 0.1% Triton × -100 in PBS for 15 min. The cells were then incubated for 1 h in blocking buffer (1% BSA in PBS) followed by incubation overnight with primary antibodies followed by secondary antibody staining for 2 h. The cells were mounted with Vectashield antifade mounting medium with DAPI (Vector Laboratories, Burlingame, CA, USA). Images were acquired using an LSM710 confocal microscope (Zeiss, Oberkochen, Germany) through a 63× oil fluorescence objective.

#### 4.10.2. IncuCyte-Live Cell Imaging

Six-well plates (Costar Flat Bottom, Corning Inc., Corning, NY, USA) were coated with 0.1% (*w/v*) poly-L-lysine (Sigma-Aldrich) 1.5 × 10^6^ cells were seeded in a 6-well plate (Corning Costar Flat Bottom) coated with poly-L-lysine. After 5 min, solution was aspirated and the surface was thoroughly rinsed with sterile tissue culture grade water. The plates were allowed to dry at least 2h before introducing cells and medium. Cells (50,000 cells/well) were seeded for overnight and the plate was taken to a 5% CO_2_ incubator with IncuCyte S3 imager installed (Sartorius, Essen BioScience, Ann Arbor, MI, USA). Changes in the cellular morphology was continuously visualized using the IncuCyte^®^ Live Cell Imaging System. This is an automated detection of percent confluency every 2h which enables real-time, live cell counting within a standard cell culture incubator.

### 4.11. Proximity Ligation Assay (PLA)

For PLA, the initial step of harvesting cells and fixation were the same as those used for immunofluorescence. Following fixation, DuoLink PLA was performed as per manufacturer’s protocol (Sigma). The primary antibodies were incubated overnight at 4 °C and are as follows: ASC (SantaCruz) + NLRP3 (Cell Signaling Technologies, Beverly, MA, USA) and controls containing individual antibodies or no antibodies. Images were acquired using a Zeiss LSM710 confocal microscope through a 63× oil fluorescence objective.

### 4.12. Flow Cytometry

L363 cells (2 × 10^6^ cells/well) seeded in 6-well plates were exposed to various concentrations of D089. For apoptosis assays, 2 × 10^6^ cells were washed twice with cold PBS and cells were analyzed with either Annexin V-PI Apoptosis Detection Kit I (Catalog #: 556547, BD Biosciences, San Jose, CA, USA) or Annexin-V-FITC (Catalog #:556420, BD) and 7-AAD (Catalog #:559925, BD). Briefly, cells were collected, washed with PBS and stained with FITC-Annexin V and propidium (PI)/7-AAD staining in the 1× Annexin V Binding Buffer. The samples were subsequently analyzed by flow cytometry within 1 h. Unstained cells, cells stained exclusively with FITC-Annexin V, PI or 7-AAD were used as controls for compensations of the lasers. The stained cells were analyzed by flow cytometry (BD FACS Calibur) within 1 h. Data were analyzed with FlowJo v7.6.5 software (Treestar, Ashland, OR, USA). The mitochondrial membrane potential (MMP) (ΔΨ_m_) was assessed using tetramethylrhodamine methyl ester (TMRM, ThermoFisher Scientific, Catalog # I34361) detection followed by normalization with Mito Tracker Green (MG) (ThermoFisher Scientific, Catalog # M7514) which explicitly stains mitochondria regardless of the changes in the MMP. Briefly, cells treated with D089 15 μM for 48 h and were washed and then incubated with TMRM (100nM) and MG (100 nM) for 30 min. The fluorescence of 10,000 cells was measured by flow-cytometry (BD LSRFortessa Cell Analyzer). Data were analysed using FlowJo. Mitochondria-targeted MitoSOX™ Red fluorogenic dye (M36008; Thermo Fisher Scientific; Rockford, IL, USA) was used to measure mitochondrial superoxide accumulation according to the manufacturer’s instructions after 2 h of D089 treatment. The fluorescence of 10,000 cells was measured by flow-cytometer (BD LSRFortessa Cell Analyzer). Data were analysed using FlowJo v7.6.5 software.

### 4.13. LDH Assay

LDH activity in cell supernatants was assessed using LDH-Quant-Cytotoxicity Assay Kit II (ThermoFisher). L363 cells were plated in 96-well plates before exposure to D089 or solvent control (DMSO) for 8 h. The percentage of LDH release was calculated as follows: % LDH release = (sample LDH activity − background LDH)/(total LDH activity − background LDH) × 100.

### 4.14. Quantitative ELISA for Human Interleukin-1 Beta (hu IL-1β)

L363 cells were treated with DMSO or D089 for 8 h from the cell culture supernatants using hu IL-1β ELISA kit (ThermoFisher Scientific). The human IL-1β solid-phase sandwich ELISA (enzyme-linked immunosorbent assay) is designed to measure the amount of the IL1-β bound between a matched antibody pair. Briefly, IL-1β monoclonal antibody conjugated to biotin was pre-coated in the wells of the supplied microplate. Samples, standards, or controls are then incubated with the immobilized antibody. The sandwich is formed by the addition of the Streptavidin-HRP conjugate, used as second antibody. A TMB (tetramethyl-benzidine) substrate solution is added that reacts with the HRP-streptavidin-biotin-IL1-β-complex to produce measurable signal. The intensity of this signal is directly proportional to the concentration of IL1-β present in the sample. A standard curve was generated and used to calculate IL1-β concentrations (pg/mL) in culture media.

### 4.15. Statistical Analysis

Values were analyzed with GraphPad Prism 6 and presented as the mean ± SD. Unpaired student’s t-tests were performed to compare the differences between groups. *p* < 0.05 was considered statistically significant.

## 5. Conclusions

The relationship between pyroptosis and cancer is context dependent and complex. Pyroptosis may be capable of being either pro- or anti-cancer [71]. On one hand, the release of inflammatory mediators and induction of hyperactive signaling pathways may promote tumorigenesis and lead to drug resistance. The primary evidence for this alternative is that high levels of certain gasdermins involved in pyroptosis have been associated with poor prognosis in certain types of cancers [71]. On the other hand, pyroptosis is a PCD mechanism that can inhibit the incidence and progression of cancer. Drugs and fatty acids (e.g., ivermectin, zinc-oxide nanoparticles, docosahexaenoic acid) that induce this form of PCD have been effective in treating cancer models [71]. Many cancers, including myeloma, frequently escape apoptosis through the upregulation of anti-apoptotic proteins such as bcl2, bclxl and Mcl1 [72]; therefore, pyroptosis induction provides an alternate therapeutic option. The potential to combine drugs that induce cell death via different mechanisms has recently gained attention [73,74]. Thus, our studies have identified a new small molecule, the benzofuran containing MYC G4 stabilizer D089 which can be used for further studies of pyroptosis and senescence in cancer models.

## Figures and Tables

**Table 1:** Reactome pathways enriched. Top reactome pathways identified by the PANTHER over-representation test for genes differentially regulated after 8 h D089 treatment (see Table S1 for the list of differentially expressed genes). (FDR: False discovery Rate).

Reactome Pathways	Raw (*p* Value)	FDR
The NLRP1 inflammasome (R-HAS-844455)	9.04 × 10^−5^	2.30 × 10^−3^
TFAP2 (AP-2) family regulates transcription of cell cycle factors (R-HSA-8866911)	1.89 × 10^−4^	4.36 × 10^−3^
BH3-only proteins associate with and inactivate anti-apoptotic BCL-2 members (R-HSA-111453)	4.34 × 10^−6^	1.86 × 10^−4^
SOS-mediated signaling (R-HSA-112412)	2.52 × 10^−4^	5.51 × 10^−3^
Activated NTRK3 signals through PI3K (R-HSA-9603381)	2.52 × 10^−4^	5.46 × 10^−3^
Activation of the AP-1 family of transcription factors (R-HSA-450341)	7.49 × 10^−6^	2.93 × 10^−4^

**Table 2:** siRNAs used for RNA Interfernce.

siRNA	Catalog Number
siGENOME Human CASP3 siRNA-SMARTpool	M-004307-02-0010
siGENOME Human CASP1 siRNA-SMARTpool	M-004401-03-0010
siGENOME Non-Targeting siRNA Pool	D-001206-14-50

**Table 3:** Primers used for real-time quantitative PCR.

Primers	Forward	Reverse
MYC	TGAGGAGACACCGCCCAC	CAACATCGATTTCTTCCTCATCTTC
DDIT3	CAGAACCAGCAGAGGTCACA	AGCTGTGCCACTTTCCTTTC
B2M	GCTATCCAGCGTACTCCAAAG	GCTGAAAGACAAGTCTGAATG

**Table 4:** Antibodies used for immunoblotting and immunofluorescence.

Antibodies	Company	Catalog Number
c-Myc	Abcam	ab-32072
p16 for Immunofluorescence	Cell Signaling Technology	92803
p16 for Immunoblotting	SantaCruz Biotechnologies	sc-1661
p21 for Immunofluorescence	Cell Signaling Technology	2947
γH2AX for Immunofluorescence	Cell Signaling Technology	9718
p-EIF2α	Cell Signaling Technology	3398
EIF2α	Cell Signaling Technology	9722
PERK	Cell Signaling Technology	5683
p-PERK	Cell Signaling Technology	3179
XBP1s	Cell Signaling Technology	12782
ATF4	Cell Signaling Technology	11815
DDIT3/CHOP	Cell Signaling Technology	2895
IRE1α	Cell Signaling Technology	3294
NFE2L2/NRF2	Cell Signaling Technology	12721
Grp78/Bip/ HSP5A	Cell Signaling Technology	3177
Caspase 1	Cell Signaling Technology	2225
Caspase 3	Cell Signaling Technology	9662
Caspase 8	Cell Signaling Technology	9746
Caspase 9	Cell Signaling Technology	9502
GSDMD	Cell Signaling Technology	97558
NLRP3	Cell Signaling Technology	13158
GAPDH	Cell Signaling Technology	5174
ASC	SantaCruz Biotechnologies	sc-514414
PANX1	Cell Signaling Technology	91137
HMGB1	Cell Signaling Technology	6893
NFκB	SantaCruz Biotechnologies	sc-8008
Vincullin	Abcam	ab18058
β-actin	Cell Signaling Technology	3700
Mouse IgG (H+L)	Invitrogen, ThermoFisher Scientific	62-6520
Rabbit IgG (H+L)	Invitrogen, ThermoFisher Scientific	31460
Alexa Fluor 488 (anti-mouse)	Abcam	ab150113
Alexa Fluor 488 (anti-rabbit)	Abcam	ab150077

## Supplementary Materials

The following are available online at https://www.mdpi.com/2072-6694/12/10/2952/s1, Figure S1:D089 mediated cytotoxicity in MM cells leads to senescence and cell death, Figure S2: D089 does not induce cell death in cells expressing c-Myc independent of G4 regulation, Figure S3: D089 triggers pyroptosis by inducing cleaved caspase 1, 8 and Gasdermin D, Table S1: D089 responsive genes after treatment (normalized gene expression >14 (50th percentile threshold; at least 1.7 log2 fold(FC) (70th percentile)), Table S2: Original Blots with molecular markers.
