# Supplementary material for: A Small Molecule Stabilizer of the MYC G4-Quadruplex Induces Endoplasmic Reticulum Stress, Senescence and Pyroptosis in Multiple Myeloma
Source: Cancers (Basel). 2020 Oct 13;12(10):2952. doi: 10.3390/cancers12102952 (PMC7650714; doi:10.3390/cancers12102952)
Supplement: Supplementary file 1 [file cancers-12-02952-s001.zip › cancers-935985-supplementary/cancers-935985 Figure S1 and S2.pdf]

Supplementary Materials: A Small Molecule Stabilizer of the MYC G4-Quadruplex Induces Endoplasmic Reticulum Stress, Senescence and Pyroptosis in Multiple Myeloma

Snehal M. Gaikwad, Zaw Phyo, Anaisa Quintanilla Arteaga, Sayeh Gorjifard, David R. Calabrese, Daniel Connors, Jing Huang, Aleksandra M. Michalowski, Shuling Zhang, Zheng-Gang Liu, John S. Schneekloth, Jr. and Beverly A. Mock

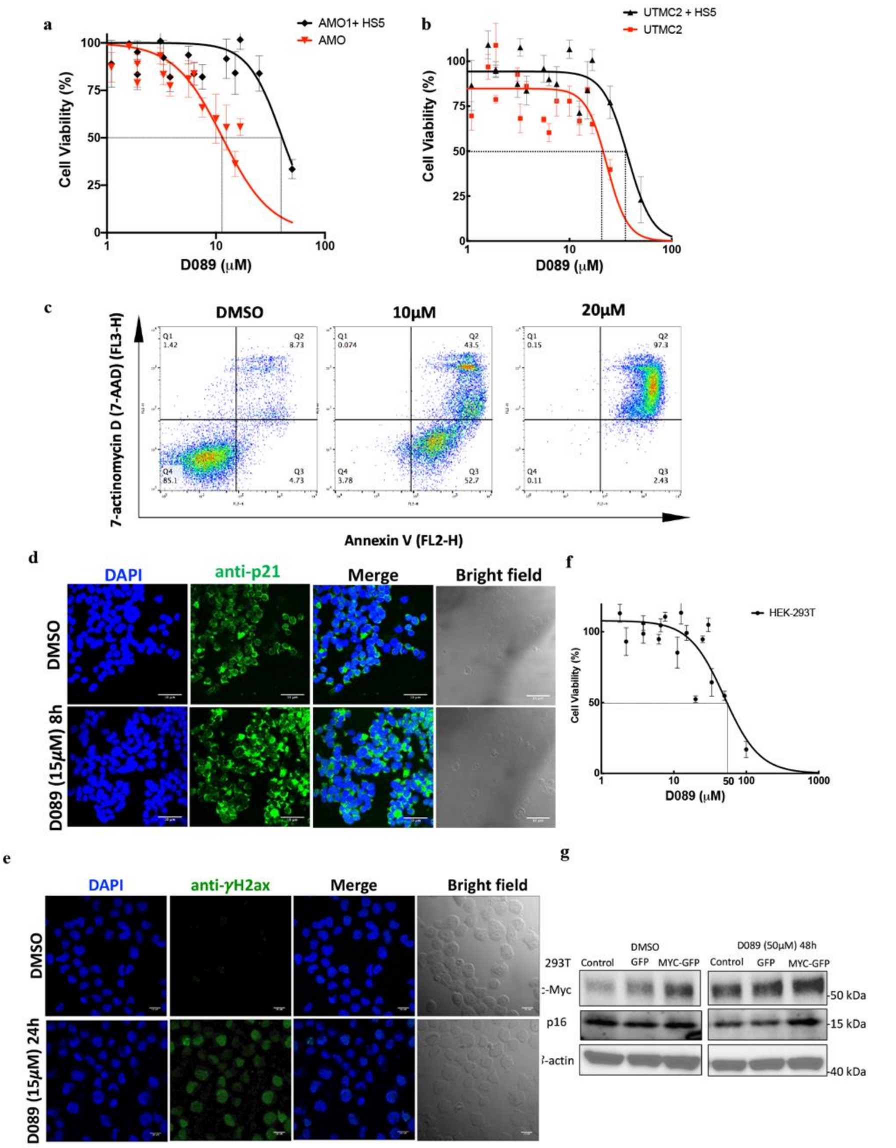

Figure S1. D089 mediated cytotoxicity in MM cells leads to senescence and cell death. (**a**,**b**) Cell viability of MM cell-lines AMO1 and UTMC2 in co-culture with HS-5- BMSCs after 48h of treatment with D089 (MTS assay). The data is presented as percentage of cell viability relative to untreated cells. Cytotoxicity of D089: for AMO1 cells either alone [(IC_{50}) = 14 (11–16) μM] or in co-culture with BMSCs [IC_{50} = 44 (37–52) μM]. Similarly, for UTMC2 cells either alone [(IC_{50}) = 23 (20–27) μM] or in co-culture with BMSCs [IC_{50} = 37 (30–47) μM]. (**c**) L363 cells were stimulated with different concentrations of D089 for 24 h and cell death was assessed with annexin-V and 7-AAD staining followed by flow cytometry. The double-positive staining in quadrant 2 (Q2) represents the percentage of cell death;

(**d,e**) L363 cells were treated with 15 μM D089 for 8 h and both p21 (d) and g-H2AX (e) protein expression were analyzed by confocal microscopy (p21 and g-H2AX in green); cells were counter stained with DAPI to detect nuclei (blue). Scale bars: 10 μm; Magnification: 63× objective lens. (**f**) Dose response of HEK293T cells assessed after 48 h of treatment with D089. The data are presented as percent of viable cells relative to untreated cells and the IC_{50} concentration was 50 μM. (**g**) Immunoblotting of HEK293T cell lysates transiently transfected with CMV-c-MYC-IRES-GFP followed by D089 (50 μM) treatment for 24 or 48 h.

a

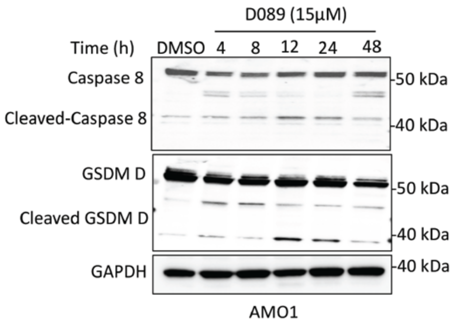

b

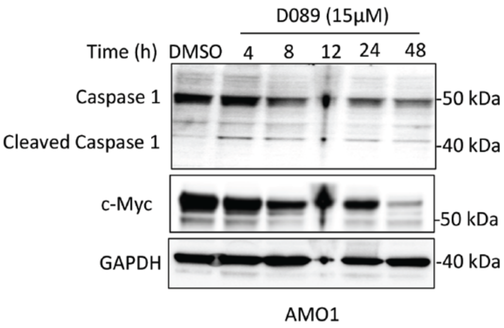

Figure S2. D089 triggers pyroptosis by inducing cleaved caspase 1, 8 and Gasdermin D. Immunoblotting of CASP8 and GSDM D (**a**) and MYC and CASP1 (**b**) after D089 (15 μM) treatment in AMO1 cells over 48 h. Cleaved products in both indicate active protein.

© 2020 by the authors. Licensee MDPI, Basel, Switzerland. This article is an open access article distributed under the terms and conditions of the Creative Commons Attribution (CC BY) license (http://creativecommons.org/licenses/by/4.0/).
